# Characterization of synovial angiogenesis in osteoarthritis patients and its modulation by chondroitin sulfate

**DOI:** 10.1186/ar3771

**Published:** 2012-03-12

**Authors:** Cécile Lambert, Marianne Mathy-Hartert, Jean-Emile Dubuc, Eulàlia Montell, Josep Vergés, Carine Munaut, Agnès Noël, Yves Henrotin

**Affiliations:** 1 Bone and Cartilage Research Unit, Institute of pathology, CHU Sart-Tilman, 4000 Liège, Belgium; 2 Orthopaedic Department, Cliniques Universitaires St Luc, 1200 Brussels, Belgium; 3 Pharmacological Research Unit, Scientific Medical Department, Bioibérica, S.A, 08029 Barcelona, Spain; 4 Laboratory of Tumor and Developmental Biology, GIGA-Cancer, Institute of Pathology, University of Liège, 4000 Liège, Belgium

## Abstract

**Introduction:**

This work aimed at comparing the production of inflammatory and pro- and anti-angiogenic factors by normal/reactive (N/R) or inflammatory (I) areas of the osteoarthritic synovial membrane. The effects of interleukin (IL)-1β and chondroitin sulfate (CS) on the expression of pro- and anti-angiogenic factors by synovial fibroblasts cells (SFC) were also studied.

**Methods:**

Biopsies from N/R or from I areas of osteoarthritic synovial membrane were collected at the time of surgery. The inflammatory status of the synovial membrane was characterized by the surgeon according to macroscopic criteria, including the synovial vascularization, the villi formation and the hypertrophic aspect of the tissue. We assessed the expression of CD45, von Willebrand factor and vascular endothelial growth factor (VEGF) antigen by immunohistochemistry in both N/R and I biopsies. The production of IL-6, -8, VEGF and thrombospondin (TSP)-1 by N/R or I synovial cells was quantified by ELISA. SFC were cultured in the absence or in the presence of IL-1β (1 ng/ml) and with or without CS (10, 50, 200 μg/ml). Gene expression of pro-angiogenic factors (VEGF, basic fibroblast growth factor (bFGF), nerve growth factor (NGF), matrix metalloproteinase (MMP)-2 and angiopoietin (ang)-1) and anti-angiogenic factors (vascular endothelial growth inhibitor (VEGI), TSP-1 and -2) were determined by real time RT-PCR. Production of VEGI and TSP-1 was also estimated by ELISA.

**Results:**

Immunohistochemistry showed the increase of lymphocyte infiltration, vascular density and VEGF expression in I compared to N/R synovial biopsies. Synovial cells from I areas produced more IL-6, IL-8 and VEGF but less TSP-1 than cells isolated from N/R synovial biopsies. The expression of pro-angiogenic factors by SFC was stimulated by IL-1β. A time dependent regulation of the expression of anti-angiogenic factor genes was observed. IL-1β stimulated the expression of anti-angiogenic factor genes but inhibited it after 24 h. CS reversed the inhibitory effect of IL-1β on anti-angiogenic factors, VEGI and TSP-1.

**Conclusions:**

We demonstrated that synovial biopsies from I areas expressed a pro-angiogenic phenotype. IL-1β induced an imbalance between pro- and anti-angiogenic factors in SFC and CS tended to normalize this IL-1β-induced imbalance, providing a new possible mechanism of action of this drug.

## Introduction

Osteoarthritis (OA) is an important cause of pain and disability in the ageing population. The main features of OA include articular cartilage degradation, subchondral bone abnormal remodeling and synovial membrane inflammation leading to joint swelling, synovitis and pain [1]. Angiogenesis and inflammation of synovial membrane are closely integrated processes in OA pathogenesis [2,3] but it remains largely unclear how inflammation and angiogenesis are cross-regulated.

We hypothesized that the angiogenesis of the synovial membrane results of an imbalance between pro-angiogenic and anti-angiogenic factors and that interleukin (IL)-1β, one of the most potent cytokine in OA physiopathology, may induce this imbalance. More precisely, we have investigated the expression of pro-angiogenic (Vascular Endothelial Growth Factor (VEGF), basic Fibroblast Growth Factor (bFGF), Nerve Growth Factor (NGF), Angiopoietin (Ang)-1 and Matrix Metalloproteinase (MMP)-2) and anti-angiogenic factors (Vascular Endothelial Growth Inhibitor (VEGI), Thrombospondine (TSP)-1 and -2) by synovial fibroblast cells (SFC) treated by IL-1β. VEGF and bFGF are potent and extensively studied pro-angiogenic factors [4]. They induce proliferation, sprouting and tube formation of endothelial cells. NGF, acting in concert with VEGF, also plays a role as a pro-angiogenic factor [5]. Ang-1 is a critical mediator of the late stages of vessel development. It maintains and stabilizes mature vessels by binding to an endothelium-specific receptor, Tie2 [6]. By promoting the breakdown of the endothelial basement membrane, MMP-2 allows the migration of endothelial cells and the formation of primitive vessels into avascular regions of tissue [7]. TSP-1 and -2 are among the first anti-angiogenic factors identified. They act by blocking endothelial cell proliferation, migration and apoptosis, by reducing the availability of angiogenic factors and by regulating the extracellular proteases [8]. VEGI, also known as tumor necrosis factor superfamily member 15 (TNFSF15) or TNF ligand related molecule 1 (TL1), is a cytokine with potent anti-angiogenic properties principally studied in cancer as an inhibitor of endothelial cell proliferation and tumor growth [9].

VEGF [10], bFGF [11], NGF [12], Ang-1 [13] and MMP-2 [14] are overexpressed in the synovial membrane of RA patients compared with either normal or OA patients. Anti-angiogenic factors, TSP-1 [15], TSP-2 [16] and VEGI [17], are also up-regulated in RA synovial membrane.

Chondroitin sulfate (CS) is a major component of the extracellular matrix of cartilage implicated in its elasticity and its resistance to loading. CS is one of the most used molecules in the management of OA and numerous clinical trials and meta-analyses have shown its clinical benefits to decrease pain, improve functional disability and reduce non-steroidal anti-inflammatory drugs (NSAIDs) or acetaminophen consumption [18-22]

Although several mechanisms of action have been described for this drug [23,24], the exact mechanism(s) of action of this molecule still remains an exciting field of investigation [25]. Taking into account the reduction in swelling and joint effusion produced by CS on synovitis of knee OA patients described in the GAIT study [26], the evaluation of the possible effect of CS in the angiogenic processes observed in OA synovial membrane should lead at least to a better knowledge of its mechanisms of action and may represent a major advance in the demonstration of its efficacy.

To provide insight into the network between inflammation and angiogenesis, we have compared inflammatory (immunohistochemical staining for lymphocytes) and angiogenic (immunohistochemical staining for VEGF and for von Willebrand factor) status of OA synovial membrane biopsies from normal or reactive (N/R) or inflammatory (I) areas of OA knee. We have investigated the relationship between inflammatory markers (IL-6 and IL-8) and angiogenic markers (VEGF and TSP-1) in synovial cells isolated from these distinct areas of synovial membrane. We have further studied the effects of IL-1β on the expression of pro- and anti-angiogenic factors by SFC. The modulation of these factors by CS was also investigated.

Understanding the regulatory molecular mechanisms of angiogenesis in OA synovial membrane should lead to novel OA therapies which would selectively inhibit the undesirable angiogenesis. The demonstration of anti-angiogenic action of CS could provide a novel therapeutic approach for OA targeting.

## Materials and methods

### Patients

Synovial tissue samples were obtained from 16 patients (10 women, 6 men; mean age 67 years, range 54 to 89 years) with OA of the knee at the time of total knee joint replacement surgery. All the subjects provided their informed consent and the ethic approval (Ethic committee agreement of Catholic University of Louvain, n°: B403201111664) was granted for this study. For each patient, biopsies were selected according to the macroscopic aspect of the synovial membrane [27,28]:

1. Normal synovial membrane (grade 0):

✓ Few translucent, slender villi with a fine vascular network can be clearly seen

✓ Proliferation of opaque villi

2. Reactive synovial membrane (grade 0.5):

✓ Villi have normal morphology or somewhat thicker and squat appearance

✓ Vascular network not seen due to loss translucence

3. Inflammatory synovial membrane (grade 1):

✓ Hypervascularization of synovial membrane and/or proliferation of hypertrophic and hyperemic villi are apparent

### Histology and immunohistochemistry of synovial membrane

Synovial biopsies were fixed, dehydrated in xylene and embedded in paraffin under standard procedures. Five μm sections were cut and deparaffinized and stained with haematoxylin and eosin (HE) according to standard protocol. Lymphocytes were identified by immunohistochemitry using anti-CD45 antibody (dilution 1/100; M 0701, Dako, Heverlee, Belgium). Blood vessels were visualized using anti-von Willebrand factor antibody (dilution 1/200; A 0082, Dako, Heverlee, Belgium). Sections were also stained with anti-VEGF (dilution 1/50; sc-152, Santa Cruz Biotechnology, Heidelberg, Germany) antibody. Secondary antibodies, horseradish peroxidase enzyme conjugated and 3,3'-diaminobenzidine (DAB) substrate, were used to develop immunostaining according to manufacturer's instructions. Slides were counterstained with HE.

### Isolation and culture of synovial cells

N/R and I synovium biopsies were separately digested with collagenase from Clostridium histolyticum, Type IA (1 mg/ml) (Sigma-Aldrich, Bornem, Belgium) in complete medium (CM) (DMEM (Dulbecco's Modified Eagle's Medium) supplemented with 10 mM HEPES, 100 U/ml penicillin, 100 μg/ml streptomycin, 2 mM glutamine and 10% fetal calf serum (Lonza, Verviers, Belgium)) for four hours at 37°C. The cell suspensions were passed through a 70-μm filter to remove any undigested tissue. The filtered cell suspensions were then collected by centrifugation at 800 g. Synovial cells (SC) from either N/R or I biopsies (N/R SC or I SC) were cultured in a 12-well plate at 2 × 10^5 ^cells per well in 1 ml of CM, at 37°C in a 5% CO_2 _humidified atmosphere. After two days, medium was changed to eliminate non adherent cells and cultivated in fresh medium for five days. Synovial fibroblast cells (SFC) were obtained and used after four passages of SC in culture flasks.

### Treatment of synovial fibroblast cells

IL-1β and CS were tested on SFC. Cells were seeded into a six-well plate at a density of 1 × 10^5 ^cells/well in 2 ml of CM. When the cells reached confluence, the CM was replaced with 1% serum media for 24 h to render the cells quiescent. Cells were incubated in the same medium with or without CS (10, 50, 200 μg/ml). CS (CS Bio-*Active*^®^, Bioibérica, Barcelona, Spain) used in this study was from bovine origin with a disaccharide sulfation profile of 63% of 4-sulfated, 31% of 6-sulfated and 6% of O-sulfated. When IL-1β (1 ng/ml) (Roche Diagnostics, Vilvoorde, Belgium) was used, cells were pre-incubated with CS for 1 h before the addition of IL-1β.

### DNA assay

Cells were homogenized in 0.5 ml of phosphate buffered saline (PBS) (Lonza, Verviers, Belgium) by ultrasonic dissociation for 15 s at 4°C. DNA content was measured in the cell extracts using a fluorimetric method of Labarca and Paigen [29].

### Immunoassays for IL-6, IL-8, VEGF, TSP-1 and VEGI

IL-6, IL-8, VEGF, TSP-1 and VEGI were determined in the culture supernatant by ELISA (Biosource Europe, Merelbeke, Belgium for IL-6 and IL-8; RD System, Abingdon, UK for VEGF and TSP-1; PeproTech, London, UK for VEGI) using specific monoclonal and polyclonal antibodies according to the manufacturers' protocols. The production of the different molecules was expressed per μg of DNA. The results were presented as the percent of production from N/R areas.

### RNA extraction and Real Time Reverse Transcriptase-Polymerase Chain Reaction (RT PCR)

Total RNA were extracted using the RNeasy Mini Kit (Qiagen, Venlo, Netherlands) and were reverse transcribed with SuperScript III Reverse Transcriptase (Invitrogen, Merelbeke, Belgium) according to the manufacturer's instructions. The cDNAs were quantified by real-time quantitative PCR using the LightCycler 480 instrument (Roche Diagnostics, Vilvoorde, Belgium) and the SYBR Premix Ex Taq kit (Takara, Verviers, Belgium). The level of gene expression was determined by interpolation with a standard curve. To standardize mRNA levels, GAPDH, a house keeping gene, was amplified as an internal control. Gene expression was normalized by calculating the ratio between the number of cDNA copies of the desired gene and that of GAPDH. The oligonucleotide primers sequences were as follows: GAPDH forward, 5'-TTGGTATCGTGGAAGGACTCA-3'; GAPDH reverse, 5'-TGTCATCATATTTGGCAGGTTT-3'; VEGF forward, 5'-TGCCTTGC TGCTCTAC-3'; VEGF reverse, 5'-CACACAGGATGGCTTGAA-3'; bFGF forward, 5'-TCAAAGGAGTGTGTGCTAACC-3'; bFGF reverse, 5'-TTTCAGTGCCACATACCAAC-3'; NGF forward, 5'-CATCATCCCATCCCATCT -3'; NGF reverse, 5'-GTACTGTTTG AATACACTGTTGTTAAT-3'; MMP-2 forward, 5'-CCGTCGCCCATCATCAA-3'; MMP-2 reverse, 5'-CAGTCCAAAGAACTTCTGCAT-3'; Ang-1 forward, 5'-ATTGAGTTAAT GGACTGGGAAG-3'; Ang-1 reverse, 5'-ATTGAGTTAATGGACTGGGAAG-3'; TSP-1 forward, 5'-CAGACCGCATTGGAGATAC-3'; TSP-1 reverse, 5'-CCATCGTTGTCATCA TCGTG-3'; TSP-2 forward, 5'-AATGACGGTGTGACCGA-3'; TSP-2 reverse, 5'- ACCCTCTCCATTGTTGTCT-3'; VEGI forward, 5'-CCCTTCCTTGCAGGACT-3'; VEGI reverse, 5'- CCCTTGGCTTATCTCCGT-3'. The results are represented as the percentage of gene expression in the basal condition or as the percentage of expression obtained in the absence of CS (control) depending on experimental procedures.

### Statistical analysis

Non-parametric Mann-Whitney U test was used to analyze statistical differences. Differences were considered statistically significant with *P-*value < 0.05. Correlation was performed by Spearman non-parametric analysis.

## Results

### Histological evaluation of inflammation and angiogenesis of N/R and I synovial biopsies

**Figure 1:**
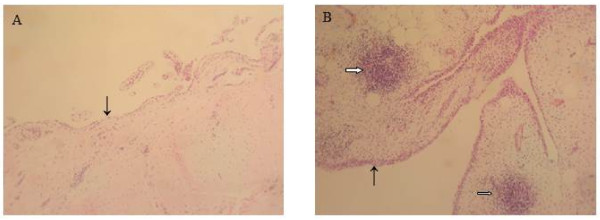
**Histological analysis of NR (A) and I (B) biopsies of synovial membrane**. At the time of surgery, synovial biopsies coming from NR or I area of synovial membrane were macroscopically selected as described in Materials and methods. Biopsies were fixed, embedded in paraffin and sectioned before staining with HE. → intima linning, ⇒ lymphoid node.

**Figure 2:**
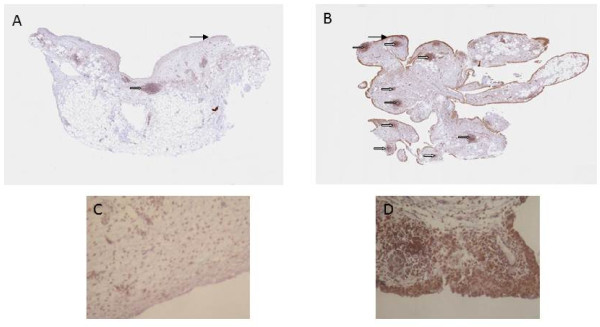
**Immunohistochemical detection of lymphocytes in N/R (A and C) and in I (B and D) synovial biopsies**. At the time of surgery, synovial biopsies coming from N/R or I areas were macroscopically selected as described in Materials and methods. Immunohistochemical staining of lymphocytes was performed using anti CD45 antibody. Positive staining was detected as brown peroxidase reaction product. (A) and (B) Global image of the section → intima linning, ⇒ lymphoid node (C) and (D) magnification (×100) of intima lining.

**Figure 3:**
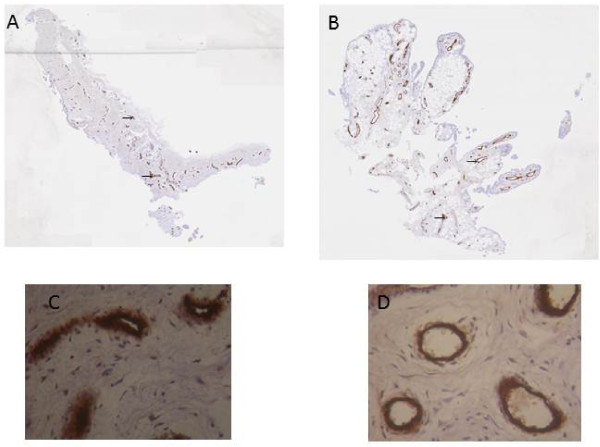
**Immunohistochemical detection of blood vessels in NR (A and C) and in I (B and D) synovial biopsies**. At the time of surgery, synovial biopsies coming from NR or I areas were macroscopically selected by as described in Materials and methods. Immunohistochemical staining of blood vessels (→) was performed using anti-von Willebrand factor antibody. Positive staining was detected as brown peroxidase reaction product. (A) and (B) global image of the section, (C) and (D) magnification (×100) of vessel lumens.

**Figure 4:**
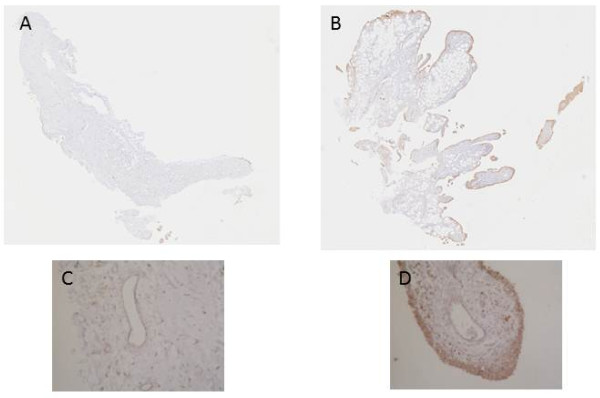
**Immunohistochemical detection of VEGF in NR (A, and C) and in I (B and D) synovial biopsies**. At the time of surgery, synovial biopsies coming from NR or I areas were macroscopically selected as described in Materials and methods. NR and I synovial biopsies were stained with anti-VEGF antibody. Positive staining was detected as brown peroxidase reaction product. (A) and (B) global image of the section, (C) and (D) magnification (×10) of intima lining and vessel lumen.

A marked increase in cellularity was observed in the intima lining of I biopsies (Figure 1) compared with N/R biopsies. The intima lining layer of I synovial biopsies was hyperplasic with multiple layers of cells while N/R synovial lining was formed by one or two cell layers. Invasive lymphoid nodes in the sublining region were present in I biopsies, but not in N/R biopsies. I biopsies were also characterized by villi formation. This histological analysis validated the sampling method of biopsies and the discrimination of N/R and I area of the synovial membrane. We have further compared CD45, von Willebrand and VEGF expression in N/R and I biopsies by immunohistochemistry. Lymphocytes were visualized using antibody against CD45, a transmembrane glycoprotein expressed by leukocytes (Figure 2). In I synovial biopsies, there was an increase in the CD45-positive cells with a predominant localization in the intima lining. To investigate the blood vessel formation in synovial membrane, we performed immunohistochemical staining using antibody against von Willebrand factor (Figure 3). Blood vessels were distributed throughout the depth of the synovial membrane without preferential distribution in the lining cells. Vascular density and vessel size were higher in I than in N/R biopsies. A staining for VEGF was observed in perivascular and sublining cells in both N/R and in I biopsies (Figure 4). An acute positive staining was observed in the intima lining layer of I but not N/R biopsies.

### Inflammatory and angiogenic factors expression by SC from N/R and I areas

The production of inflammatory and angiogenic factors by SC isolated from either N/R or I areas of the synovial membrane was compared. N/R SC produced 8,497 ± 3,598 pg/μg DNA of IL-6, and 17,726 ± 6,682 pg/μg DNA of IL-8.

**Figure 5:**
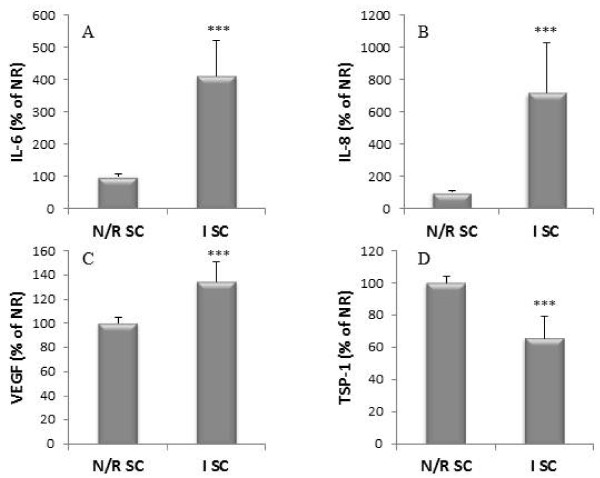
**IL-6 (A), IL-8 (B), VEGF (C) and TSP-1 (D) production by primary N/R and I SC**. SC were isolated from N/R and I synovial biopsies as described in Materials and methods. Cells were cultivated for five days and the production of proteins was measured in conditioned culture supernatants by ELISA. Productions were normalized to DNA content. Results are expressed as the percent of the production of N/R SC. Results are expressed as mean ± SEM of 10 independent experiments performed with synovial cells isolated from 10 different donors. Comparison of mean values was performed by Mann-Whitney non-parametric analysis. *** *P *< 0.001: production of I SC cells was significantly different than production of N/R SC.

IL-6 and IL-8 (Figure 5A, B) production by I SC was significantly higher than those of N/R SC (for IL-6: 411 ± 110% of N/R SC production, *P *< 0.001; for IL-8: 716 ± 310% of N/R SC production, *P *< 0.001). I SC produced more VEGF (134 ± 16% of N/R SC production, *P *< 0.001, Figure 5C) and less TSP-1 (66 ± 14% of N/R SC production, *P *< 0.001, Figure 5D) than N/R SC.

In addition, we assessed the correlation between inflammatory cytokines (IL-6, IL-8) and angiogenic factors (VEGF, TSP-1). VEGF production showed a strong positive correlation with IL-6 and IL-8 (r = 0.67, *P *< 0.0001 and r = 0.65, *P *< 0.0001, respectively). A significant negative correlation was obtained between TSP-1 and IL-8 (r = -0.4, *P *= 0.002). The correlation between TSP-1 and IL-6 was at the limit of the statistical significance (r = -0.25, *P *= 0.06).

### Effect of IL-1β on mRNA expression of pro-/anti-angiogenic factors by SFC

**Figure 6:**
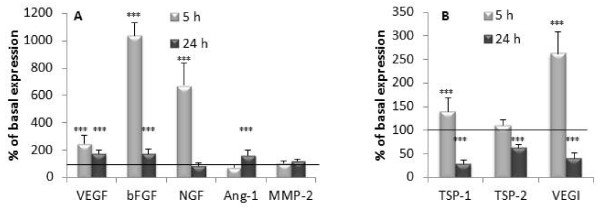
**Effect of IL-1β on pro (A) and anti-angiogenic (B) factor gene expression**. FSC were cultured for 5 or 24 h in the absence or in the presence of IL-1β (1 ng/ml). Total RNA was isolated in cellular extract and pro-angiogenic (VEGF, bFGF, NGF, Ang-1 and MMP-2) (A) and anti-angiogenic (TSP-1, TSP-2 and VEGI) (B) mRNA expression was analysed by real time RT PCR. Results are expressed as a percentage of the basal condition (cells incubated without IL-1β). Each condition was tested in duplicate. Results are expressed as mean ± SEM of three independent experiments.

SFC were incubated in the absence (basal condition) or in the presence of IL-1β (1 ng/ml) during 5 or 24 h. The gene expression of five pro-angiogenic factors (VEGF, bFGF, NGF, Ang1 and MMP-2) (Figure 6A) and three anti-angiogenic factors (VEGI, TSP-1 and TSP-2) (Figure 6B) was analyzed by real time RT PCR.

After 5 h of incubation, the expression of VEGF, bFGF and NGF genes was increased by IL-1β (VEGF: 250 ± 58 of basal expression, *P *< 0.001; bFGF: 1,032 ± 99% of basal expression, *P *< 0.001; NGF: 672 ± 161% of basal expression, *P *< 0.001). After 24 h, the stimulating effect of IL-1β on VEGF and bFGF genes was less marked (respectively 173 ± 30% of basal expression, *P *< 0.001 and 175 ± 34% of basal expression, *P *< 0.001). No significant effect of IL-1β was obtained for NGF gene expression after 24 h of treatment. Ang-1 gene expression was not modified by IL-1β after 5 h of treatment while a weak but significant increase was observed after 24 h (159 ± 37% of basal expression, *P *< 0.001). IL-1β did not influence the MMP-2 gene expression.

Figure 6B showed the effect of IL-1β on mRNA expression of anti-angiogenic factors. For TSP-1 and VEGI, a biphasic time-dependent effect of IL-1β was observed: a stimulation after 5 h of treatment (TSP-1: 140 ± 28% of basal expression, *P *< 0.01; VEGI: 262 ± 47% of basal expression, *P *< 0.001) and an inhibition after 24 h of treatment (TSP-1: 29 ± 8% of basal expression, *P *< 0.001; VEGI: 41 ± 10% of basal expression, *P *< 0.001). A time-dependent biphasic effect of IL-1β was also recorded on the production of TSP-1 and VEGI proteins. After 5 h of treatment, IL-1β stimulated TSP-1 (130.2 ± 6.65% of basal expression, *P *< 0.01) and VEGI (140.2 ± 10.1% of basal expression, *P *< 0.001) while it inhibited TSP-1 and VEGI production after 24 h (TSP-1: 71.3 ± 5.2% of basal expression, *P *< 0.01; VEGI: 77.5 ± 4.23% of basal expression, *P *< 0.001). After 5 h of treatment, IL-1β did not modify TSP-2 gene expression while an inhibition was observed after 24 h (62 ± 8% of basal expression, *P *< 0.001, Figure 6B).

### CS modulation of pro-/anti-angiogenic factor expression in SFC

Using real time RT-PCR technology, we investigated the effects of CS on pro- and anti-angiogenic factors. SFC were incubated for 5 and 24 h with increasing concentrations of CS (10, 50, 200 μg/ml) and with or without IL-1β (1 ng/ml). In all tested conditions, CS did not modify the basal expression of pro- (VEGF, bFGF, NGF, Ang-1 and MMP-2) and anti-angiogenic (VEGI, TSP-1 and 2) genes (data not shown). The stimulating effect of IL-1β on the expression of pro-angiogenic genes was unaffected by CS (data not shown).

**Figure 7:**
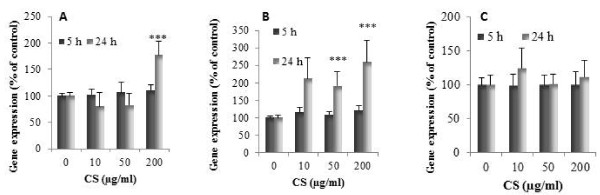
**Effect of CS on IL-1β-dependent anti-angiogenic gene expression**. SFC were pre-incubated with CS (10, 50, 200 μg/ml) for 1 h. IL-1β (1 ng/ml) was added to culture medium for 5 or 24 h incubation. Total RNA was isolated in SFC and VEGI (A), TSP-1 (B) and TSP-2 (C) mRNA expression was analyzed by real time RT PCR. Results are expressed as a percentage of the control (cells incubated without CS). Each condition was tested in duplicate. Results are expressed as mean ± SD of five independent experiments. Comparison of mean values was performed by Mann-Whitney non-parametric analysis. ***P < 0.001: CS treated group was significantly different from control.

**Figure 8:**
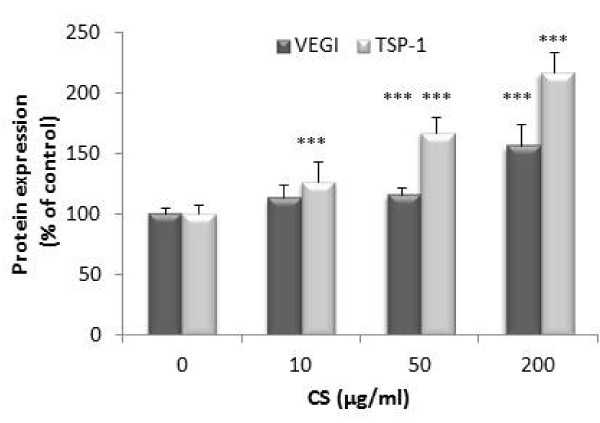
**Effect of CS on IL-1β-dependent VEGI and TSP-1 production**. SFC were pre-incubated with CS (10, 50, 200 μg/ml) for 1 h. IL-1β (1 ng/ml) was added to culture medium for 24 h. TSP-1 and VEGI were measured in conditioned culture supernatants by ELISA. Results are normalized to DNA content. Results are expressed as a percentage of the control (cells incubated without CS). Each condition was tested in triplicate. Results are expressed as mean ± SD of three independent experiments. Comparison of mean values was performed by Mann-Whitney non-parametric analysis. ***P < 0.001: CS treated group was significantly different from control.

After 5 h of treatment, CS did not induce significant changes in the IL-1β-dependent up-regulation of gene expression of anti-angiogenic factors (data not shown). Figure 7 showed the effect of CS on the inhibitory effect of IL-1β on the expression of VEGI, TSP-1 and TSP-2 genes observed after 24 h of treatment. After 24 h, VEGI mRNA levels were enhanced in the presence of 200 μg/ml of CS (177 ± 26% of control, *P *< 0.001). TSP-1 gene expression was significantly increased by 50 μg/ml of CS (191 ± 41.2% of control, *P *< 0.001) and by 200 μg/ml of CS (259.5 ± 61% of control, *P *< 0.001) of CS. No significant variation of TSP-2 was obtained. At the concentration of 50 and 200 μg/ml, CS partially reversed the inhibitory effect of IL-1β on VEGI protein production (Figure 8). Indeed, a significant increase of VEGI production was observed with 50 μg/ml of CS (115 ± 5% of control, *P *< 0.001). This increase was significantly marked at 200 μg/ml of CS (156 ± 17% of control, *P *< 0.001). Similarly, CS reversed the inhibitory effect of IL-1β on TSP-1 production. CS increased TSP-1 production in a dose dependent manner (r = 0.82, *P *< 0.001).

## Discussion

Synovial inflammation is directly implicated in the initiation and progression of OA [1]. Uncontrolled angiogenesis is a critical event of synovial inflammation and these two interdependent processes, that is, angiogenesis and inflammation, are now considered as important contributors of OA [2,30].

The mechanism leading to the crosstalk between angiogenesis and inflammation is still insufficiently clear. To contribute to the better understanding of this crosstalk in OA synovial membrane, we have developed an original methodology comparing inflammation and angiogenesis in synovial membrane with different grades of synovitis. We used the Ayral's synovitis scores [27] to select, in the same OA synovial membrane, biopsies coming from N/R or I synovial areas. This method of biopsy sampling was validated by the histological and immunohistochemical analyses. Our results supported those of Haywood *et al. *[2], who showed that the endothelial cells density and VEGF immunoreactivity of OA synovial membrane increased with inflammation grade and macrophage infiltration. This study contributed to showing the concerted evolution of inflammation and angiogenesis in OA synovial membrane.

We have shown a difference of production of pro-inflammatory cytokines (IL-6 and IL-8), pro-angiogenic factor (VEGF) and anti-angiogenic factor (TSP-1) between N/R and I primary SC. These results suggested a shift in the balance of angiogenic factors in favor of the development of neovascularization. IL-6 and IL-8 are the most abundant cytokines found in the synovial fluid of patients with OA and they are considered to play a central role in synovial inflammation [28]. In OA patients, IL-6 production in synovial fluid is correlated with plasma C-reactive protein and with synovial inflammatory cells infiltration [31]. VEGF is considered as a dominant pro-angiogenic factor which acts early in angiogenesis inducing an increase in blood vessel permeability and proliferation and migration of endothelial cells. VEGF has been associated with synovial angiogenesis [2]. The inhibitory effect of TSP-1 on angiogenesis is largely described [8]. As an inhibitor of angiogenesis, TSP-1 overexpression decreases inflammation and blood vessel density in synovial membrane. It also reduces cartilage lesions in rats where OA is induced by anterior cruciate ligament transection [32]. In addition, VEGF production showed a strong positive correlation with IL-6 and IL-8, while significant negative correlation was obtained between TSP-1 and IL-8. These results highlight the role played by VEGF and TSP-1 in the link between inflammation and angiogenesis of the synovial membrane.

Synovial inflammation is an important source of pro-inflammatory mediators. IL-1β produced by synovial cells induces a degradative cascade leading to joint damage. In this work, we have studied the modulation of gene expression of pro-angiogenic (VEGF, bFGF, NGF, Ang-1, MMP-2) and anti-angiogenic (VEGI, TSP-1 and 2) factors by IL-1β in SFC. IL-1β increased the level of mRNA on pro-angiogenic factors. VEGF, bFGF and NGF were rapidly enhanced after 5 h of treatment, while Ang-1 increased after 24 h suggesting a delayed involvement of Ang-1 in angiogenesis. Our findings are in complete agreement with previous studies demonstrating the up-regulation of VEGF and NGF gene expression in SFC by IL-1β [33,34]. Using Northern blot analysis, Gravalesse *et al. *[35] reported that TNF-α but not IL-1β stimulated mRNA of Ang-1 in RA SFC. This discrepancy can be explained by the differences in the origin of the cells and by the method used to study gene expression. More surprising was the time-dependent regulation of anti-angiogenic VEGI and TSP-1 gene expression. A stimulating effect of IL-1β was observed after 5 h but an inhibitory effect after 24 h. This complex regulation suggests a cascade of indirect and time dependent events which negatively regulate VEGI and TSP-1 production. A transitory IL-1β-dependent molecule expression could be responsible for the early stimulation of VEGI and TSP-1. The disappearance of this molecule with time could explain the loss of the stimulating effect observed after 5 h. In addition, the inhibitory effect could be linked to the presence of a late IL-1β-dependent molecule expression. Little information is accessible on the regulation of VEGI, TSP-1 and -2 by IL-1β. The down-regulation of TSP-1 was reported in human endothelial cells and rabbit chondrocytes [36,37] but never in SFC. Interestingly, a time dependent phorbol-myristate-acetate (PMA) regulation of VEGI expression (stimulation for 6 h, inhibition for 24 h) was described in endothelial cells [38]. This observation supports our result showing a biphasic effect of IL-1β on VEGI gene expression.

Our data suggest that after 24 h of treatment, IL-1β induced an imbalance between pro- and anti-angiogenic factors production in SFC in favor of pro-angiogenic status and by this way could promote synovial membrane angiogenesis.

Finally, in the present study we examined the effect of CS on the expression of pro- and anti-angiogenic factors in OA SFC. Although CS is currently used in the management in OA, its mechanism of action has not been yet fully clarified. Anti-inflammatory properties of CS were largely described but less is known about its effect on angiogenesis [39]. Macrophage migration on endothelium is one of the key steps for angiogenesis [40]. Macrophages promote angiogenesis by the synthesis of angiogenic factors including VEGF and by the remodeling of the extracellular matrix. An *in vitro *study has demonstrated that CS acted as an inhibitor of monocytes THP-1 cell migration suggesting that CS could prevent angiogenesis [41]. Our results reveal that CS counteracts the IL-1β-dependent TSP-1 and VEGI down-regulation at both gene and protein levels. This finding could suggest an anti-angiogenic effect of CS.

## Conclusions

For the first time, we demonstrated the presence of pro-inflammatory and pro-angiogenic pathways in the I areas of synovial membrane compared to N/R. IL-1β induced an imbalance between pro- and anti-angiogenic factors in SFC and CS tended to normalize this IL-1β-induced imbalance. Since angiogenesis is a key process in OA progression, our data suggest that the beneficial role of CS in OA management could, at least in part, derive from its anti-angiogenic properties.

## Abbreviations

Ang: angiopoietin; bFGF: basic Fibroblast Growth Factor; CM: complete medium; CS: chondroitin sulfate; DAB: diaminobenzidine; DMEM: Dulbecco's Modified Eagle's Medium; ELISA: enzyme-linked immunoabsorbent assay; HE: haematoxylin and eosin; I: inflammatory; IL: interleukin; MMP: matrix metalloproteinase; NGF: nerve growth factor; N/R: normal/reactive; NSAIDs: non-steroidal anti-inflammatory drugs; OA: osteoarthritis; PBS: phosphate buffered saline; PMA: phorbol-myristate-acetate; SC: synovial cells; SFC: synovial fibroblast cells; TL1: TNF ligand related molecule 1; TNFSF15: tumor necrosis factor superfamily member 15; TSP: thrombospondin; VEGF: vascular endothelial growth factor; VEGI: vascular endothelial growth inhibitor.

## Competing interests

EM is the Head of the Medical Area from Bioibérica, SA. JV is Medical Director of Bioibérica, SA. YH received a grant from Bioibérica, SA to carry out this project. The authors declare that they have no competing interests.

## Authors' contributions

CL and MM participated in the study design, acquisition of data, interpretation of results and manuscript preparation. J-ED helped in collecting and processing samples. EM and JV provided CS and helped design the study. CM and AN performed the histological experiments. YH conceived and coordinated the project. All authors were involved in revising of the manuscript and approved the final manuscript.
